# Comparative analysis of aggressiveness in giant cell tumor of bone between upper and lower extremities: A systematic review and meta-analysis

**DOI:** 10.1016/j.jbo.2025.100663

**Published:** 2025-02-08

**Authors:** Rhyan Darma Saputra, Dita Anggara Kusuma, Fathih Kaldani, Khoirul Fahmi

**Affiliations:** a Orthopaedic and Traumatology Department, Faculty of Medicine, Sebelas Maret University, Indonesia; b Orthopaedic and Traumatology Department, Moewardi General Hospital, Indonesia

**Keywords:** Giant cell tumor of bone (GCTB), Aggressiveness, Upper Extremity, Lower Extremity, Recurrence

## Abstract

**Background and Objective:**

Giant cell tumor of bone (GCTB) is among the most prevalent benign primary bone tumors, characterized by its potential for aggressive local recurrence, soft tissue invasion, and, though rare, lung metastasis. Emerging evidence suggests unique behavioral patterns of GCTB in extremities. This study seeks to rigorously compare the aggressiveness of GCTB in the upper versus lower extremities, centering on recurrence rates.

**Method:**

This systematic review and meta-analysis, conducted in accordance with PRISMA guidelines, sourced data from MEDLINE/PubMed, Cochrane, Scopus, CINAHL/EBSCO, and reference lists of pertinent studies. Two independent reviewers screened studies, with discrepancies resolved by discussion. Eligible studies included a minimum of 10 participants. Data extraction and analysis were performed by an additional team of two researchers.

**Results:**

Out of 1,283 studies spanning from 1984 to 2023, 30 met eligibility, encompassing 2,672 participants. The mean age was 32.77 ± 12.99 years, with an average follow-up of 75.53 ± 65.88 months. GCTB predominantly affected the lower extremities, accounting for 1,937 cases. Notably, comparisons of aggressiveness between upper and lower extremity GCTB revealed no statistically significant difference (OR = 1.10, p = 0.56 for Surgery Group; OR = 1.16, p = 0.45 for Local Adjuvant Group; and OR = 1.71, p = 0.32 for Drug/Denosumab Group).

**Conclusion:**

This analysis underscores the lower extremities as the primary site for GCTB but finds no significant difference in aggressiveness between upper and lower extremities. These findings challenge assumptions about GCTB behavior based on tumor location and highlight the need for further investigation to fully elucidate the complex biology of extremity GCTB.

## Highlights

• GCTB in the upper and lower extremities shows comparable aggressiveness.
• Anatomical challenges in the upper extremities, such as the distal radius, may create a perception of higher aggressiveness.
• Local adjuvants effectively reduce recurrence rates without requiring distinct protocols based on tumor location.
• Preoperative denosumab increases recurrence risk if curettage is not meticulously performed, especially in high-grade lesions.
• MSTS scores are higher for upper extremities, likely due to easier functional criteria and the ability to preserve hand function even after aggressive surgery.

## Graphical abstract

**Figure:**
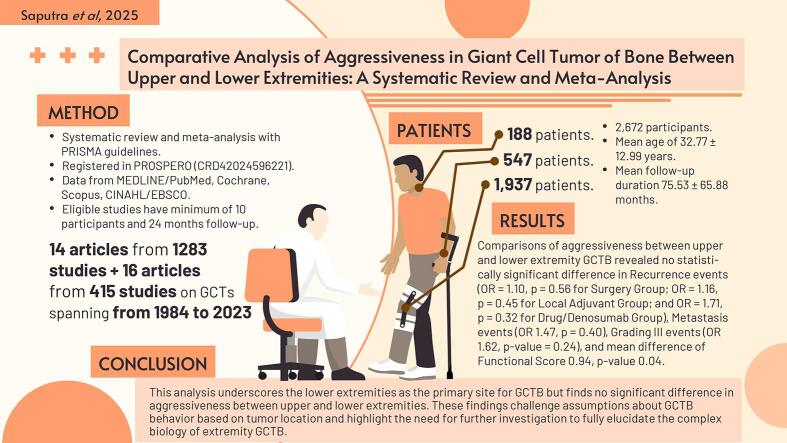

## 1 Introduction

Giant cell tumor of bone (GCTB) is among the most common benign tumors, initially described by Cooper and Travers in 1818 [1]. GCTB accounts for approximately 5 % of all primary bone tumors and 20 % of benign bone tumors [2]. Predominantly affecting young adults aged 20–40, GCTB shows a slight female predominance [3]. It typically occurs in the metaphyseal or epiphyseal regions of long bones, most often the distal femur, proximal tibia, and distal radius [4]. Although GCTB is usually benign, it demonstrates unpredictable behavior, including local aggressiveness, bone and cortical destruction, soft tissue expansion, local recurrence, metachronous lesions, and, rarely, metastasis [5]. The risk of metastasis is higher in younger patients, those with stage III tumors, and in cases with local recurrence [6], [7].

Some studies suggest that GCTB in the upper extremities is more aggressive than in the lower extremities [8], [9], [10]. Whereas others indicate that GCTB is more common and aggressive in the lower extremities [11], [12], [13]. Despite these findings, a comprehensive synthesis comparing GCTB aggressiveness in the upper versus lower extremities is still lacking. This study aims to address this gap by investigating the aggressiveness of GCTB in these regions, focusing on local recurrence and metastasis. Understanding the behavior of GCTB across different anatomical sites, particularly in the upper versus lower extremities, is crucial for optimizing management strategies and improving patient outcomes.

## 2 Methods

This systematic review and *meta*-analysis was conducted following PRISMA guidelines, the study is registered in PROSPERO (CRD42024596221) and structured on the PI/ECO framework.

### 2.1 Literature search strategy

This review was carried out using four major databases—PubMed/MEDLINE, EBSCO/CINAHL, Scopus, and Cochrane—covering studies up to August 2024. Parameters include: Population, patients with giant cell tumor of bone (GCTB) in the extremities; Intervention/Exposure, upper extremity involvement; Comparison, lower extremity involvement; Outcome, oncological measures like recurrence, tumor grading, metastasis, and functional outcomes assessed by the Musculoskeletal Tumor Society (MSTS) score. Searches were conducted using precise keywords below, tailored for each database, with search results saved in PMID and RIS formats for organized review.

1. **PubMed (1996**–**2024)**: ((giant cell tumor[Title/Abstract]) OR (giant cell tumour[Title/Abstract]) OR (osteoclastoma[Title/Abstract]) OR (GCT[Title/Abstract])) AND ((upper[Title/Abstract]) OR (extremity[Title/Abstract]) OR (lower[Title/Abstract]) OR (hand[Title/Abstract]) OR (shoulder[Title/Abstract]) OR (ankle[Title/Abstract]) OR (foot[Title/Abstract])) AND ((aggressiveness[Title/Abstract]) OR (severity[Title/Abstract])).
2. **Scopus (1941**–**2024)**: TITLE-ABS-KEY((giant AND cell AND tumo*) OR (gct) OR (osteoclastoma)) AND ((aggressiv*) OR (sever*)) AND ((extremit*) OR (upper) OR (lower) OR (hand) OR (shoulder) OR (ankle) OR (foot)).
3. **Cochrane Database (2000**–**2024)**: ((giant cell tumo*) OR (osteoclastoma) OR (GCT)) AND ((aggressiv*) OR (sever*)) AND ((upper) OR (lower) OR (hand) OR (shoulder) OR (ankle) OR (foot)).

**EBSCO (1999**–**2024)**: ((giant cell tumo*) OR (osteoclastoma) OR (GCT)) AND ((aggressiv*) OR (sever*)) AND ((upper) OR (lower) OR (hand) OR (shoulder) OR (ankle) OR (foot)).

### 2.2 Study selection

The review’s inclusion criteria encompassed clinical studies of GCTB in both upper and lower extremities, with over 10 participants and a minimum follow-up of 24 months across various treatment modalities. Studies exclusively focused on upper or lower extremities alone, unclear outcome comparison data, non-English publications, or inaccessible full texts were excluded.

Search results were saved in PMID and RIS formats and subsequently reviewed using Rayyan software to screen for duplicates, abstracts, and full-text eligibility, with two independent reviewers (FK and KF) conducting the review. A third reviewer (RDS) was consulted to resolve any disagreements and ensure consensus.

### 2.3 Risk of bias

Eligible studies underwent rigorous risk of bias assessment by two researchers (FK and KF) using the ROBINS-I and ROBINS-E tools. Any discrepancies were discussed between researchers until resolved. Data visualization was designed using Robvis Tool.

### 2.4 Data extraction

Data extraction was centered on comparing recurrence rates, tumor grading, and metastasis as key oncological outcomes, alongside the MSTS score as a measure of functional outcomes, between GCTB cases in the upper and lower extremities. These parameters were chosen to evaluate GCTB aggressiveness, consistent with methodologies employed in previous retrospective studies [11], [13], [16], [32]. Data extraction was performed by two independent researchers (DAK and RDS) using WPS Office Free Version 12.2.0. The dataset included participant demographics, tumor characteristics, the number of GCTB cases in the upper and lower extremities, recurrence events, metastasis occurrences, grade III tumor incidences, treatment strategies, follow-up durations, and MSTS scores.

Subsequently, the extracted data were analyzed using R Studio 2024.09.0 to conduct a detailed *meta*-analysis, which included calculating oncological outcome using odds ratios, determining mean MSTS scores, and generating forest plots. Reporting bias was assessed through a funnel plot, and in cases of asymmetry, Egger’s test was employed to evaluate the significance of potential reporting bias.

Subgroup analyses provided deeper insights, examining recurrence rates through odds ratios across three categories: surgical intervention, the application of local adjuvants during curettage, and the preoperative use of denosumab. Additional subgroup analyses explored metastasis rates and the prevalence of Campanacci grade III tumors, comparing odds ratios across groups. Functional outcomes were assessed by analyzing mean MSTS scores, delivering a comprehensive understanding of GCTB’s impact across anatomical sites and treatment approaches.

## 3 Results

**Fig. 1:**
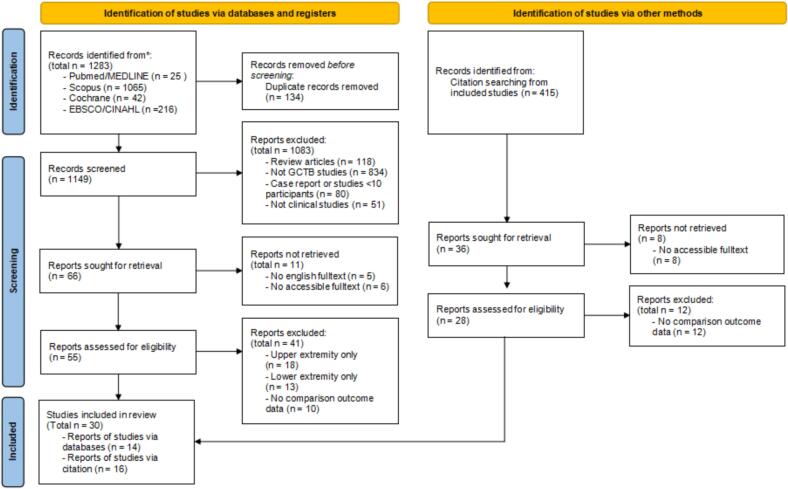
PRISMA diagram of study selection.

Initial search through PubMed/MEDLINE, EBSCO/CINAHL, Scopus, and Cochrane databases identified 1,283 records. After duplicate removal using Rayyan software, 1,149 unique records remained. Subsequently, 1,094 studies were excluded for not meeting the inclusion criteria, lacking full-text availability, or being unavailable in English version. Following a thorough full-text review of the remaining 55 studies led to the exclusion of 41 studies due to unclear comparative outcomes between the upper and lower extremities or inclusion of participants from only single extremity group, leaving 14 studies. A citation review of references from these 14 articles identified an additional 415 studies, of which 16 met the eligibility criteria (Fig. 1).

In total, 30 relevant studies from database searches and citation reviews were assessed for risk of bias using ROBINS-I for interventional studies and ROBINS-E for retrospective observational studies. Of these, 14 studies were rated as having a moderate risk of bias, while 16 were categorized as having a high risk of bias. Despite variations in bias risk, all 30 studies were included in the *meta*-analysis due to the limited availability of eligible studies for subgroup analysis.

The eligible studies (n = 30), spanning from 1984 to 2023, represented a total of 2,672 participants (Table 1). The participants had a mean age of 32.77 ± 12.99 years and a mean follow-up duration of 75.53 ± 65.88 months. Among the GCTB cases analyzed, 547 patients were located in the upper extremities, 1,937 in the lower extremities, and 188 in axial sites. In the upper extremities, the distal radius was the most frequently affected site, with 289 cases, whereas in the lower extremities, the distal femur was the most common site, numbered 826 cases, followed by the proximal tibia with 665 cases. These anatomical distribution findings align with expectations. Of the 30 eligible studies, 17 retrospectively focused on recurrences after all treatment modalities, 9 emphasized recurrences following the use of local adjuvants, and the remaining 4 focused on preoperative use of denosumab.

**Table 1:** Eligible studies from databases and citation reviews.

No	Study	Mean Age	Upper	Lower	Treatment	Subgroup	Outcome
1	Levine, 1984	30.41 ± 11.98	3	14	6 Currettage, 12 Resection, 3 Amputation	Surgery	Rec, Gra
2	Lausten, 1996	35.45 ± 12.85	8	16	18 Currettage, 13 Resection	Surgery	Rec, Met, Gra
3	Oda, 1998	32 ± 12.01	9	32	28 Currettage, 19 Resection	Surgery	Rec, Met, Gra, MSTS
4	Biscaglia, 2000	27.4 ± 13.25	8	21	19 Currettage, 6 Resection, 1 Amputation	Surgery	Rec, Gra
5	Saiz, 2004	34 ± 10.5	6	34	40 Currettage with local adjuvants	Local Adjuvant	Rec
6	Su, 2004	35 ± 13.25	20	56	56 Currettage, 31 Resection	Surgery	Rec
7	Lackman, 2005	40 ± 14.75	9	54	63 Currettage with local adjuvants	Local Adjuvant	Rec, MSTS
8	Balke, 2008	33.28 ± 13.04	29	147	188 Currettage with local adjuvant, 20 Resection, 2 Amputation, 4 No Surgery	Local Adjuvant	Rec, Met
9	Becker, 2008	NA	98	286	306 Currettage with local adjuvants, 78 Resection	Local Adjuvant	Rec
10	Fraquet, 2009	36 ± 9.75	2	28	30 Currettage with local adjuvants	Local Adjuvant	Rec
11	Klenke, 2011	31.1 ± 12.8	14	32	28 Currettage, 18 Resection	Surgery	Rec
12	Klenke, 2011	35.4 ± 15.8	24	65	95 Currettage, 22 Resection, 1 No Surgery	Surgery	Rec
13	Saikia, 2011	29 ± 11	51	85	77 Currettage, 45 Resection, 2 Amputation, 2 No Surgery, 15 No Information	Surgery	Rec, MSTS
14	Takeuchi, 2011	30.5 ± 16	23	87	94 Currettage, 16 Resection	Surgery	Rec
15	Yanagisawa, 2011	24.72 ± 13.18	7	4	6 Currettage, 4 Resection, 1 Amputation	Surgery	Rec, Met, Gra
16	Niu, 2012	31.4 ± 15	48	209	157 Currettage, 126 Resection	Surgery	Rec
17	Gao, 2014	31.8 ± 11.75	5	60	65 Currettage with local adjuvants	Local Adjuvant	Rec
18	Cheng, 2015	32 ± 14.5	12	67	57 Currettage, 23 Resection	Surgery	Rec
19	Takeuchi, 2016	34 ± 17.5	23	80	98 Currettage, 5 Resection	Surgery	Rec
20	Agarwal, 2018	30.22 ± 9.71	9	44	37 Currettage, 22 Resection, with Denosumab pre operatively	Drug/Denosumab	Rec, Gra
21	Scoccianti, 2018	38.5 ± 12.02	4	14	21 Currettage, with Denosumab pre operatively	Drug/Denosumab	Rec, Met
22	Chinder, 2019	29.6 ± 9.8	34	77	123 Currettage, with Denosumab pre operatively	Drug/Denosumab	Rec
23	Greenberg, 2019	33.70 ± 9.18	6	11	17 Currettage with local adjuvant	Local Adjuvant	Rec, Gra, MSTS
24	Hasan, 2019	34.34 ± 12.62	12	31	39 Currettage, 6 Resection, 1 Amputation	Surgery	Rec
25	Gillani, 2020	25.75 ± 5.74	12	28	40 Currettage with local adjuvant	Local Adjuvant	Rec
26	Sano, 2020	35.3 ± 14.75	14	52	46 Currettage, 20 Resection, with Denosumab pre operatively	Drug/Denosumab	Rec
27	Konishi, 2021	35 ± 6.57	34	154	171 Currettage, 35 Resection, 7 No Information	Surgery	Rec, Met
28	Karadeniz, 2022	36.1 ± 9.3	14	98	67 Currettage, 48 Resection, 2 No Surgery	Surgery	Rec
29	Poudel, 2022	22.32 ± 6.78	4	8	12 Currettage with local adjuvants	Local Adjuvant	Rec, Gra, MSTS
30	Abdelkawi, 2023	NA	7	45	34 Currettage, 18 Resection	Surgery	Rec, Met

Rec: Recurrence events; Met: Metastasis Events; Gra: Grade III tumor events; MSTS: MSTS Score.

### 3.1 Meta-analysis of surgical group

**Fig. 2:**
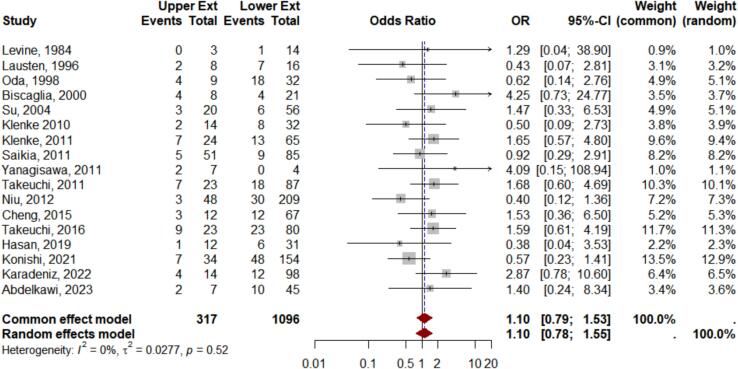
Meta analysis results of surgery subgroup.

In the surgical intervention subgroup, from 17 studies included in the *meta*-analysis, the data were found to be homogeneous, with an I^2^ test result of 0 %, τ^2^ = 0.027, p = 0.52. Similarly, the Funnel plot analysis revealed relatively symmetrical results, indicating minimal publication bias. The Forest plot analysis showed no significant difference in the comparison of GCTB aggressiveness between the upper and lower extremities, with an OR 1.10, 95 % CI 0.79–1.53, z = 0.57, p-value = 0.56 (Fig. 2).

### 3.2 Meta-analysis of local adjuvant group

**Fig. 3:**
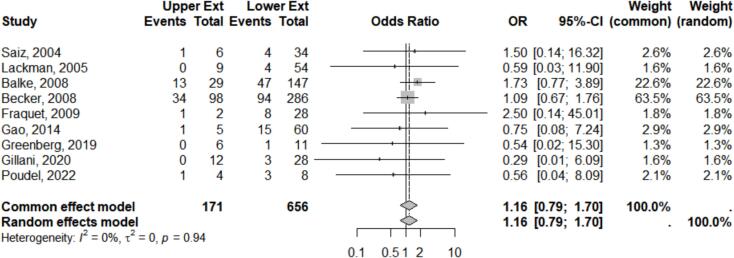
Meta analysis results of local adjuvant subgroup.

In the subgroup analysis of local adjuvant administration during curettage, both extensive and intralesional, 9 homogeneous studies were included (Fig. 3). The Funnel plot analysis also showed relatively symmetrical results I^2^ = 0 %, τ^2^ = 0.00, p = 0.94.

The *meta*-analysis in the Forest plot demonstrated no significant difference in the comparison of GCTB aggressiveness between the upper and lower extremities, with OR 1.16, 95 % CI 0.79–1.70, z = 0.74 and p = 0.45.

### 3.3 Meta-analysis of drug therapy group

**Fig. 4:**
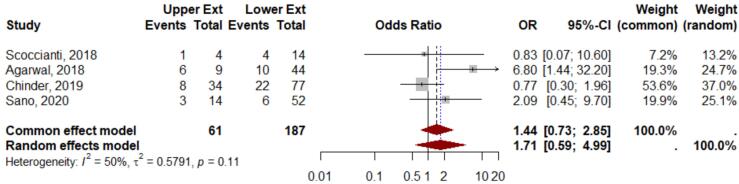
Meta analysis results of drug therapy subgroup.

While, in the subgroup analysis of preoperative Denosumab usage across 4 studies, the OR was 1.71, 95 % CI 0.59–4.99, z = 0.98, and p = 0.32, indicating also no significant difference in the comparison of GCTB aggressiveness between the upper and lower extremities (Fig. 4). The heterogeneity test indicated fairly heterogenous data, with an I^2^ 50 %, τ^2^ = 0.57, p = 0.11. The Although the funnel plot analysis showed asymmetry, the Egger’s test resulted p-value = 0.56, indicating no publication bias. The observed asymmetry in the funnel plot was likely due to heterogeneity among the studies included in the analysis.

### 3.4 Meta-analysis of metastatic events

**Fig. 5:**
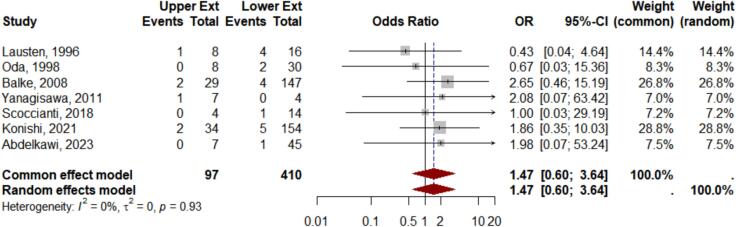
Meta analysis results of metastasis events.

Metastatic data were reported in only 8 studies, limiting the analysis to this subset. The *meta*-analysis yielded an OR 1.47, 95 % CI 0.60–3.64, z = 0.84, and p = 0.40, when comparing the aggressiveness of giant cell tumor of bone (GCTB) between the upper and lower extremities (Fig. 5). This result suggests that there is no statistically significant difference in the likelihood of metastasis between tumors located in the upper versus lower extremities. Although the number of studies is limited, the heterogeneity test results indicating a sufficient level of homogeneity, I^2^ 0 %, τ^2^ = 0.00, p = 0.93. The funnel plot analysis reveals asymmetry, with the Egger’s test showing no significant result, p-value = 0.37, suggesting differences in study design among the included studies.

### 3.5 Meta-analysis of tumor grading

**Fig. 6:**
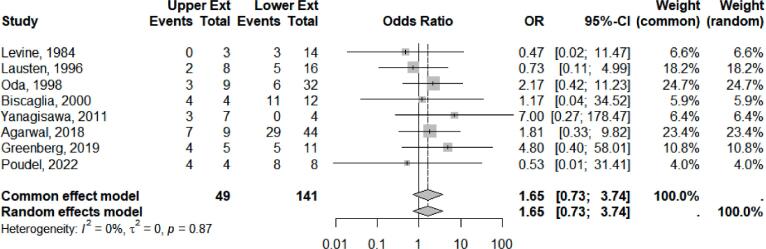
Meta analysis results of aggressiveness comparison based on grade III tumor.

Comparative analysis data on the aggressiveness of upper versus lower extremity GCTB regarding tumor grading based on Campanacci or Enneking Classification (Grade III) were obtained from 8 studies (Fig. 6). The *meta*-analysis results indicate that no significant association between GCTB upper and lower with aggressiveness according to the Campanacci III classification, OR 1.62, 95 % CI 0.71–3.69, z = 1.15, and p-value = 0.24. The heterogeneity test shows a relatively homogeneous set of studies with I^2^ 0 %, τ^2^ = 0.00, p = 0.88. The funnel plot analysis demonstrates asymmetric distribution, while Egger’s test revealed no significant p-value = 0.76, indicating differences in study design and population of the included studies.

### 3.6 Functional outcome (MSTS Score)

**Fig. 7:**
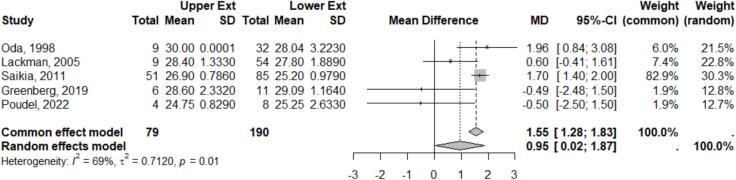
Meta analysis results of MSTS Score comparison.

Only 5 studies provided comparative data on MSTS Scores for upper versus lower extremity GCTB. The combined mean MSTS Score for the upper extremity was 27.44 ± 1.60, and for the lower extremity 26.64 ± 2.37. Meta-analysis of the means from each study showed slightly but significant difference favoring upper extremity, with the mean difference 0.94, 95 % CI 0.02–1.87, and p-value 0.04 (Fig. 7). However, because of the small number of included studies and limited data, the heterogeneity test revealed high degree of variability, I^2^ 69 %, τ^2^ = 0.71, p = 0.01. This finding was supported by funnel plot analysis that demonstrates asymmetrical plot indicating publication bias due to limited studies that provide the MSTS Score data.

## 4 Discussion

This *meta*-analysis encompassed 30 studies conducted over nearly four decades (1984–2023), including a total of 2,672 participants. The analysis aimed to test the hypothesis that giant cell tumor of bone (GCTB) in the upper extremities exhibits greater aggressiveness compared to the lower extremities. Subgroup analyses of recurrence rates were performed across various treatment modalities, including surgical intervention, local adjuvant therapy, and preoperative denosumab administration. Additional subgroup analyses evaluated metastasis, tumor grading, and functional outcomes as measured by the MSTS score [13], [14], [15], [16], [17], [18], [19], [20], [21], [22], [23], [24], [25], [26], [27], [28], [29], [30], [31], [32], [33], [34], [35], [36], [37], [38], [39], [40], [41], [42].

The overall analysis revealed that GCTB in the upper and lower extremities demonstrated relatively comparable outcomes. This finding aligns with prior studies, which concluded that the tumor's location is not a definitive prognostic factor for aggressiveness, including recurrence, metastasis, and tumor grading [14], [16], [25], [27].

While the overall analysis did not identify statistically significant differences, the odds ratios slightly favored the upper extremity. Several studies have highlighted that GCTB in the upper extremity, particularly in challenging locations like the distal radius, may appear more aggressive due to management challenges. These include the difficulty of achieving adequate curettage in anatomically complex and narrow regions [16], [18], [42]. Agarwal et al. supported this by noting that GCTB in the distal radius and other challenging locations is particularly difficult to manage with curettage alone [15], [36].

The comparison of recurrence rates between surgical groups for GCTB in the upper and lower extremities showed no significant differences, suggesting that the choice of surgical method need not be dictated by tumor location. Instead, it should be based on surgical preference and individual case requirements. Intralesional curettage remains the most common procedure for Grade I and II GCTB, despite its higher recurrence rates [11], [15], [16], [27], [30]. Wide resection, although associated with lower recurrence, is typically reserved for Grade III lesions, extensive bone destruction, or cases where joint salvage is not feasible [15], [27]. However, wide resection often leads to reduced functional outcomes due to the removal of critical neuromusculoskeletal structures [31], [32], [33], [39].

Some studies have suggested that higher recurrence rates after curettage may result from inadequate surgical technique of the surgical team [23], [25], [43]. However, Tsukamoto et al. found no significant difference in recurrence rates between surgeries performed by experienced and less experienced oncologic surgeons, emphasizing the importance of meticulous and comprehensive curettage with clear margins [44]. Greater awareness and precision during curettage are crucial for improving outcomes [16], [17], [38].

Similarly, no significant differences in recurrence rates were observed among groups using local adjuvants during curettage. This suggests that distinct protocols for local adjuvants are unnecessary based on tumor location. Local adjuvants such as PMMA cement, phenol, cryotherapy, and high-speed burring have been shown to reduce recurrence rates [16], [17], [23], [27], [29], [34], [36]. PMMA, in particular, enhances stability and reduces 8recurrence through its thermal and toxic effects on residual tumor cells [17], [22], [28], [29], [36], [46]. The combination of high-speed burring and PMMA results in improved outcomes in terms of both recurrence and functional stability [16], [17], [24]. However, when used in subchondral regions, PMMA may induce degenerative arthritis [21], [34], which can be mitigated by combining it with bone grafts [16], [17], [21], [45]. Techniques such as the longitudinal sandwich method proposed by Poudel et al. offer enhanced stability but still exhibit recurrence challenges [34].

In the drug therapy group receiving preoperative denosumab, no significant differences in recurrence rates were observed between upper and lower extremities. However, denosumab's preoperative use has been associated with increased recurrence risks due to the formation of small, hidden tumor pockets that may be overlooked during curettage [15], [20], [37], [38]. As such, meticulous and extensive curettage is especially critical in high-grade lesions and younger patients undergoing denosumab therapy [15], [20], [38].

When comparing aggressiveness based on tumor grading, no significant differences were observed between upper and lower extremities, consistent with previous studies [15], [18], [30]. The slightly higher odds ratio for upper extremities may be attributed to anatomical factors, such as smaller bone cross-sections and proximity to critical structures, which increase the likelihood of soft tissue disruption [35], [47], [48].

Similarly, aggressiveness based on metastasis rates were not significantly different between groups, reinforcing the findings of some studies that tumor location is not a determinant of metastatic behavior [13], [16], [27]. Risk factors for metastasis include recurrence, tumor size exceeding 100 cm^3^, pathological fractures, and Campanacci Grade III classification [13], [14], [30], [32], [40], [41].

Interestingly, functional outcomes, as measured by MSTS scores, were higher for the upper extremity compared to the lower extremity. This discrepancy may be attributed to the less demanding functional criteria for upper extremities. Additionally, the most common site for GCTB in the upper extremity is the distal radius, where aggressive surgery typically still permits good hand function retention, where aggressive surgery often still preserves hand function [33]. In contrast, the most frequent GCTB in lower extremity are the distal femur and proximal tibia, typically result in greater functional impairment due to their impact on weight-bearing activities, resulting in lower MSTS scores [39].

## 5 Conclusions

This *meta*-analysis demonstrates that the aggressiveness of GCTB does not significantly differ between upper and lower extremities across various treatment modalities. However, the perception of greater aggressiveness in upper extremity GCTB may stem from anatomical factors, such as smaller bone cross-sections and the complexity of adjacent structures, which pose challenges in achieving clear surgical margins. These findings underscore the importance of meticulous surgical technique, particularly in challenging locations.

### 5.1 Study limitations

Several limitations warrant acknowledgment. First, none of the included studies were randomized controlled trials, potentially affecting the robustness of the *meta*-analysis findings. Second, some studies carried a high risk of bias, requiring cautious interpretation. Third, the limited number of studies and variability in study design may have influenced the analysis. Fourth, reliance on published data introduces the potential for publication bias, as suggested by asymmetrical funnel plot analyses. Lastly, anatomical differences between the upper and lower extremities such as the smaller bone cross-sections in the upper extremity, highlight the need for further research to address technical surgical challenges. Future studies with larger sample sizes, standardized treatment protocols, and extended follow-up periods are essential to validate these findings and optimize management strategies.

### CRediT authorship contribution statement

**Rhyan Darma Saputra:** Conceptualization, Formal analysis, Project administration, Supervision, Validation, Writing – review & editing. **Dita Anggara Kusuma:** Conceptualization, Formal analysis, Investigation, Methodology, Resources, Validation, Writing – review & editing. **Fathih Kaldani:** Data curation, Formal analysis, Investigation, Methodology, Resources, Software. **Khoirul Fahmi:** Data curation, Formal analysis, Investigation, Methodology, Resources, Software, Visualization, Writing – original draft.

## Declaration of competing interest

The authors declare that they have no known competing financial interests or personal relationships that could have appeared to influence the work reported in this paper.

## Data Availability

Derived data supporting the findings of this study are available from the corresponding author on request.
