# Supplementary material for: Comparative analysis of aggressiveness in giant cell tumor of bone between upper and lower extremities: A systematic review and meta-analysis
Source: J Bone Oncol. 2025 Feb 8;51:100663. doi: 10.1016/j.jbo.2025.100663 (PMC11871493; doi:10.1016/j.jbo.2025.100663)
Supplement: Supplementary Data 1 [file mmc1.docx]

| **Section and Topic** | **Item #** | **Checklist item** | **Location where item is reported** |
| --- | --- | --- | --- |
| **TITLE** | | |  |
| Title | 1 | Comparative Analysis of Aggressiveness in Giant Cell Tumor of Bone Between Upper and Lower Extremities: A Systematic Review and Meta-Analysis |  |
| **ABSTRACT** | | |  |
| Abstract | 2 | Background and Objective: Giant cell tumor of bone (GCTB) is one of the most common benign primary tumors that has potential for local recurrence, soft tissue expansion, and rarely lung metastasis. Some studies stated that GCTB in extremities has unique behavior. The aim of this study is to compare the aggressiveness of GCTB in the upper and lower extremities, focusing on recurrence rates. Method: This systematic review and meta-analysis followed PRISMA guidelines. Eligible studies were identified from MEDLINE/PubMed, Cochrane, Scopus, CINAHL/EBSCO, and reference lists of relevant articles. Two independent reviewers selected studies, and discrepancies were resolved through discussion. Clinical studies with a minimum of 10 participants were included. The other two researchers extracted and analyzed data from eligible studies. Results: 30 of 1283 studies from 1984-2023 are eligible with 2672 participants included. Mean age of participants was 32.77 ± 12.99 years old with mean follow up 75.53 ± 65.88 months. GCTB mostly occurred in lower extremities with 1937 patients. Aggressiveness comparison between the upper and lower extremities revealed no statistically significant difference (OR = 1.10, p = 0.56, for Surgery Group; OR = 1.16, p = 0.45, for Local Adjuvant Group; and OR = 1.71, p = 0,32 for Drug/Denosumab Group). Conclusion: The lower extremity is the most common site of occurrence for GCTB. The aggressiveness of GCTB in the upper and lower extremities is relatively comparable. Further review are needed to understand the behavior of GCTB in extremities. |  |
| **INTRODUCTION** | | |  |
| Rationale | 3 | Giant cell tumor of bone (GCTB) is a benign but locally aggressive primary bone tumor, frequently affecting young adults and often located in the extremities. Despite its benign classification, GCTB has a significant propensity for local recurrence and, in rare cases, metastasis, notably to the lungs. Some studies suggest that biological behavior of GCTB may differ depending on the site of occurrence, potentially influencing recurrence rates and long-term outcomes. Understanding these differences is crucial, as it could guide surgical strategies and the use of adjuvant therapies tailored to the tumor’s location. Despite these clinical implications, there has been no conclusive evidence comparing the aggressiveness of GCTB in the upper versus lower extremities. |  |
| Objectives | 4 | This study aims to analyze existing literatures on recurrence and metastasis in GCTB between the extremities, thereby contributing to improved clinical decision-making and patient outcomes. |  |
| **METHODS** | | |  |
| Eligibility criteria | 5 | The inclusion criteria for this review consist of clinical studies related to GCTB in both upper and lower extremities with more than 10 participants, a minimum follow-up period of 24 months, and various treatment modalities. Exclusion criteria include studies with participants only from upper or lower extremities, unclear outcome data, non-English articles, and those with inaccessible full-text versions. |  |
| Information sources | 6 | This systematic review was conducted using four databases—Pubmed/MEDLINE, EBSCO/CINAHL, Scopus, and Cochrane— up to August 2024, in accordance with the PRISMA guidelines. |  |
| Search strategy | 7 | The database search was conducted using the following keywords:  1.PubMed (1996-2024): ((giant cell tumor[Title/Abstract]) OR (giant cell tumour[Title/Abstract]) OR (osteoclastoma[Title/Abstract]) OR (GCT[Title/Abstract])) AND ((upper[Title/Abstract]) OR (extremity[Title/Abstract]) OR (lower[Title/Abstract]) OR (hand[Title/Abstract]) OR (shoulder[Title/Abstract]) OR (ankle[Title/Abstract]) OR (foot[Title/Abstract])) AND ((aggressiveness[Title/Abstract]) OR (severity[Title/Abstract])).  2.Scopus (1941-2024): TITLE-ABS-KEY((giant AND cell AND tumo*) OR (gct) OR (osteoclastoma)) AND ((aggressiv*) OR (sever*)) AND ((extremit*) OR (upper) OR (lower) OR (hand) OR (shoulder) OR (ankle) OR (foot)).  3.Cochrane Database (2000-2024): ((giant cell tumo*) OR (osteoclastoma) OR (GCT)) AND ((aggressiv*) OR (sever*)) AND ((upper) OR (lower) OR (hand) OR (shoulder) OR (ankle) OR (foot)).  EBSCO (1999-2024): ((giant cell tumo*) OR (osteoclastoma) OR (GCT)) AND ((aggressiv*) OR (sever*)) AND ((upper) OR (lower) OR (hand) OR (shoulder) OR (ankle) OR (foot)). |  |
| Selection process | 8 | Search results were saved in PMID and RIS formats and subsequently reviewed using Rayyan software to screen for duplicates, abstracts, and full-text eligibility, with two independent reviewers (FK and KF) conducting the review. A third reviewer (RDS) was consulted to resolve any disagreements and ensure consensus. |  |
| Data collection process | 9 | Data extraction focused on comparison of recurrence rates, tumor grading, and metastasis as oncological outcomes and MSTS Score as functional outcomes between upper and lower extremity GCTB. These outcomes were analysed to assess aggressivness of GCTB as it was used by many retrospective studies before.^11,13,16,32^ Data extraction conducted by two additional researchers (DAK and RDS) using WPS Office Free Version 12.2.0.  The data consisted of participants characteristics, tumor status, number of participants of GCTB in upper and lower extremities, number of recurrence events, metastasis events, and grade III tumor events of GCTB in upper and lower extremities, treastment modalities, follow-up duration, and MSTS scores. |  |
| Data items | 10a | Population: Patients with giant cell tumor of bone (GCTB) in the extremities; Intervention/ Exposure: Upper extremity involvement; Comparison: Lower extremity involvement; Outcome: Oncological outcomes such as recurrence, tumor grading, and metastasis measured by odds ratio; and functional outcomes measured by the Musculoskeletal Tumor Society (MSTS) score. |  |
|  | 10b | Data extraction conducted by two additional researchers (DAK and RDS) using WPS Office Free Version 12.2.0, consisted of participants characteristics, tumor status, number of participants of GCTB in upper and lower extremities, number of recurrence events, metastasis events, and grade III tumor events of GCTB in upper and lower extremities, treastment modalities, follow-up duration, and MSTS scores. |  |
| Study risk of bias assessment | 11 | Eligible studies underwent rigorous risk of bias assessment by two researchers (FK and KF) using the ROBINS-I and ROBINS-E tools. Any discrepancies were discussed between researchers until resolved. Data visualization was designed using Robvis Tool. |  |
| Effect measures | 12 | Because of limited hazard ratio and risk ratio data, the effect of recurrence events, grade III tumor, metastasis event were measured by odds ratio. While functional outcome was measured by mean of MSTS score. |  |
| Synthesis methods | 13a | Extracted data were then analyzed in R Studio 2024.09.0, enabling detailed meta-analysis of events odds ratio mean of MSTS Score, and forest plot generation.  Subgroup analyses provided deeper insights, comparing recurrence rates using odds ratios across three groups: surgical intervention, the use of local adjuvants during curettage, and preoperative administration of denosumab. Additionally, subgroup analysis assessed metastasis occurrence and Campanacci grade III tumors, comparing odds ratios between groups. Functional outcomes were evaluated using the mean of MSTS score, offering a comprehensive overview of GCTB’s impact across anatomical sites and treatment modalities. |  |
|  | 13b | The exclusion criteria for eligible studies for meta analysis has to provide clear outcome data such as number of recurrence events, metastasis events, grade III participants, and mean of MSTS score, comparing upper and lower extremities. These numbers were analysed for odds ratio, standar deviation, and meta analysis. In case, there is no clear data, the number will be extracted from analysis of total participants and recurrence events. If the data still cannot be obtained, the study will be excluded from the meta-analysis. In case, there is no data about recurrence, metastasis, and grade III tumor events, the numbers were adjusted using continuity correction formula in order to obtain the odds ratio. |  |
|  | 13c | Using R Studio, all the data about number of partipants and recurrence events were divided into thrre subgroup, surgery, local adjuvant, and drug therapy. While the data about participants and metastasis events, MSTS score comparing upper and lower extremities, were also analyzed using R Studio. The analyses were done using “meta” and “metafor” packages in R Studio. |  |
|  | 13d | Subgroup analysis for recurrence events, metastasis events, and grade III tumor events, revealed heterogeneity test <50%. From these consideration, the meta analysis for recurrence events in subgroup surgery, local adjuvant, and drug therapy, metastasis events, and grade III tumor events, using fixed-effect model meta analysis. While, meta analysis for MSTS Score using random effect model because of the hetrogeneity test revealed I^2^>50%. All the analysis were done using “meta” and “metafor” packages in R Studio. |  |
|  | 13e | This study only divide subgroup meta-analyses for recurrence events. While metastasis events, grade III tumor events, and MSTS Score, meta analysis were done using the data provided by the eligible studies. Meta-regression were not conducted because of limited data available. |  |
|  | 13f | Sensitivity analysis were not conducted because of limited data available that provided by eligible studies. |  |
| Reporting bias assessment | 14 | This study analysed reporting bias assessment using Funnel plot. If the plot revealed asymmetrical, the data will be tested using Egger’s test for conducting significant bias test arising from reporting bias. |  |
| Certainty assessment | 15 | Data extraction focused on comparison of recurrence rates, tumor grading, and metastasis as oncological outcomes and MSTS Score as functional outcomes between upper and lower extremity GCTB. These outcomes were analysed to assess aggressivness of GCTB as it was used by many retrospective studies before.^11,13,16,32^ |  |
| **RESULTS** | | |  |
| Study selection | 16a | An initial search across PubMed/MEDLINE, EBSCO/CINAHL, Scopus, and Cochrane databases identified 1,283 records. After duplicate removal using Rayyan software, 1,149 unique records were retained. Subsequently, 1,094 studies were excluded for not meeting inclusion criteria, lacking full-text access, or being unavailable in English version. Following a thorough full-text review of the remaining 55 studies, 41 were excluded due to unclear comparative outcomes between upper and lower extremities or because they only included participants from a single extremity group, leaving 14 studies. A citation review of the references from these 14 articles yielded additional 415 studies, of which 16 met the eligibility criteria (Figure 1). |  |
|  | 16b | Following a thorough full-text review of the remaining 55 studies, 41 were excluded due to unclear comparative outcomes between upper and lower extremities or because they only included participants from a single extremity group, leaving 14 studies. |  |
| Study characteristics | 17 | All the eligible studies (n=30) spanning from 1984 to 2023, represented a total of 2,672 participants (Table 1). The participants had a mean age of 32.77 ± 12.99 years and a mean follow-up duration of 75.53 ± 65.88 months. Of the GCTB cases analyzed, 547 patients were located in the upper extremities, 1,937 patients in the lower extremities, and 188 patients involved axial sites. The distal radius was the most frequently affected site in the upper extremities, accounting for 289 cases, while in the lower extremities, the distal femur was the most common site with 826 cases, followed by the proximal tibia with 665 cases. These findings provide crucial insights into the anatomical distribution and frequency of GCTB, forming a foundation for further comparative analyses. |  |
| Risk of bias in studies | 18 | Ultimately, 30 relevant studies from database searches and citation reviews were assessed for risk of bias using ROBINS-I for interventional studies and ROBINS-E for retrospective observational studies. Among these, 14 studies were rated as having a moderate risk of bias, while 16 were categorized as having a high risk of bias. Despite these variations, all 30 studies were included in the meta-analysis due to the limited availability of eligible studies for subgroup analysis. |  |
| Results of individual studies | 19 | The meta analysis outcomes for each study was undone because the recurrence events meta analysis divided to subgroup for surgery, local adjuvant, and drug therapy. |  |
| Results of syntheses | 20a | The participants had a mean age of 32.77 ± 12.99 years and a mean follow-up duration of 75.53 ± 65.88 months. Of the GCTB cases analyzed, 547 patients were located in the upper extremities, 1,937 patients in the lower extremities, and 188 patients involved axial sites. The distal radius was the most frequently affected site in the upper extremities, numbered 289 cases, while in the lower extremities, the distal femur was the most common site with 826 cases, followed by the proximal tibia with 665 cases. These anatomical distribution findings are as expected. From the 30 eligible studies, 17 studies were retrospectively focused on recurrences after all treatment modalities, 9 studies emphasized on recurrences after local adjuvants administration, and the remaining 4 studies focused on using denosumab pre operatively. |  |
|  | 20b | 1.Meta-analysis of surgical group  In the surgical intervention subgroup, from 17 studies included in the meta-analysis, the data were found to be homogeneous, with an I² test result of 0%, τ2 = 0.027, p = 0.52. Similarly, the Funnel plot analysis revealed relatively symmetrical results, indicating minimal publication bias. The Forest plot analysis showed no significant difference in the comparison of GCTB aggressiveness between the upper and lower extremities, with an OR 1.10, 95% CI 0.79-1.53, z = 0.57, p-value = 0.56 (Figure 2). |  |
|  | 20c | 6.Functional outcome (MSTS Score)  Only 5 studies provided comparative data on MSTS Scores for upper versus lower extremity GCTB. The combined mean MSTS Score for the upper extremity was 27.44 ± 1.60, and for the lower extremity 26.64 ± 2.37. Meta-analysis of the means from each study showed slightly but significant difference favoring upper extremity, with the mean difference 0.94, 95% CI 0.02 - 1.87, and p-value 0.04 (Figure 7). However, because of the small number of included studies and limited data, the heterogeneity test revealed high degree of variability, I² 69%, τ2 = 0.71, p = 0.01. This finding was supported by funnel plot analysis that demonstrates asymmetrical plot indicating publication bias due to limited studies that provide the MSTS Score data. |  |
|  | 20d | This study was not conducted sensitivity analyses to assess the robustness of the synthesized results because of limited data available. |  |
| Reporting biases | 21 | 5.Meta-analysis of tumor grading  Comparative analysis data on the aggressiveness of upper versus lower extremity GCTB regarding tumor grading based on Campanacci or Enneking Classification (Grade III) were obtained from 8 studies (Figure 6). The meta-analysis results indicate that no significant association between GCTB upper and lower with aggressiveness according to the Campanacci III classification, OR 1.62, 95% CI 0.71 - 3.69, z = 1.15, and p-value = 0.24. The heterogeneity test shows a relatively homogeneous set of studies with I² 0%, τ2 = 0.00, p = 0.88. The funnel plot analysis demonstrates asymmetric distribution, while Egger’s test revealed no significant p-value = 0.76, indicating differences in study design and population of the included studies. |  |
| Certainty of evidence | 22 | 4.Meta-analysis of metastatic events  Metastatic data were reported in only 8 studies, limiting the analysis to this subset. The meta-analysis yielded an OR 1.47, 95% CI 0.60 - 3.64, z = 0.84, and p = 0.40, when comparing the aggressiveness of giant cell tumor of bone (GCTB) between the upper and lower extremities (Figure 5). This result suggests that there is no statistically significant difference in the likelihood of metastasis between tumors located in the upper versus lower extremities. Although the number of studies is limited, the heterogeneity test results indicating a sufficient level of homogeneity, I² 0%, τ2 = 0.00, p = 0.93. The funnel plot analysis reveals asymmetry, with the Egger’s test showing no significant result, p-value = 0.37, suggesting differences in study design among the included studies. |  |
| **DISCUSSION** | | |  |
| Discussion | 23a | This meta-analysis encompassed 30 studies conducted over nearly four decades (1984–2023), including a total of 2,672 participants. The analysis aimed to test the hypothesis that giant cell tumor of bone (GCTB) in the upper extremities exhibits greater aggressiveness compared to the lower extremities. Subgroup analyses of recurrence rates were performed across various treatment modalities, including surgical intervention, local adjuvant therapy, and preoperative denosumab administration. Additional subgroup analyses evaluated metastasis, tumor grading, and functional outcomes as measured by the MSTS score.^13-42^  The overall analysis revealed that GCTB in the upper and lower extremities demonstrated relatively comparable outcomes. This finding aligns with prior studies, which concluded that the tumor's location is not a definitive prognostic factor for aggressiveness, including recurrence, metastasis, and tumor grading.^14,16,25,27^ |  |
|  | 23b | Several limitations warrant acknowledgment. First, none of the included studies were randomized controlled trials, potentially affecting the robustness of the meta-analysis findings…. Lastly, anatomical differences between the upper and lower extremities such as the smaller bone cross-sections in the upper extremity, highlight the need for further research to address technical surgical challenges. Future studies with larger sample sizes, standardized treatment protocols, and extended follow-up periods are essential to validate these findings and optimize management strategies. |  |
|  | 23c | Second, some studies carried a high risk of bias, requiring cautious interpretation. Third, the limited number of studies and variability in study design may have influenced the analysis. Fourth, reliance on published data introduces the potential for publication bias, as suggested by asymmetrical funnel plot analyses. |  |
|  | 23d | This meta-analysis demonstrates that the aggressiveness of GCTB does not significantly differ between upper and lower extremities across various treatment modalities. However, the perception of greater aggressiveness in upper extremity GCTB may stem from anatomical factors, such as smaller bone cross-sections and the complexity of adjacent structures, which pose challenges in achieving clear surgical margins. These findings underscore the importance of meticulous surgical technique, particularly in challenging locations. |  |
| **OTHER INFORMATION** | | |  |
| Registration and protocol | 24a | This systematic review and meta-analysis was conducted following PRISMA guidelines, the study is registered in PROSPERO (CRD42024596221) and structured on the PI/ECO framework. |  |
|  | 24b | This systematic review and meta-analysis was conducted following PRISMA guidelines, the study is registered in PROSPERO (CRD42024596221) and structured on the PI/ECO framework. |  |
|  | 24c | There is no any amendments to information provided at registration or in the protocol. |  |
| Support | 25 | This study did not receive any external funding. |  |
| Competing interests | 26 | The authors declare that there is no conflict of interest. |  |
| Availability of data, code and other materials | 27 | Derived data supporting the findings of this study are available from the corresponding author on request. |  |

*From:*  Page MJ, McKenzie JE, Bossuyt PM, Boutron I, Hoffmann TC, Mulrow CD, et al. The PRISMA 2020 statement: an updated guideline for reporting systematic reviews. BMJ 2021;372:n71. doi: 10.1136/bmj.n71
