# Supplementary material for: Comparative analysis of aggressiveness in giant cell tumor of bone between upper and lower extremities: A systematic review and meta-analysis
Source: J Bone Oncol. 2025 Feb 8;51:100663. doi: 10.1016/j.jbo.2025.100663 (PMC11871493; doi:10.1016/j.jbo.2025.100663)
Supplement: Supplementary Data 2 [file mmc2.docx]

**Risk of Bias Analysis**

1. Risk of bias using ROBINS-E Tool for Observational studies

**
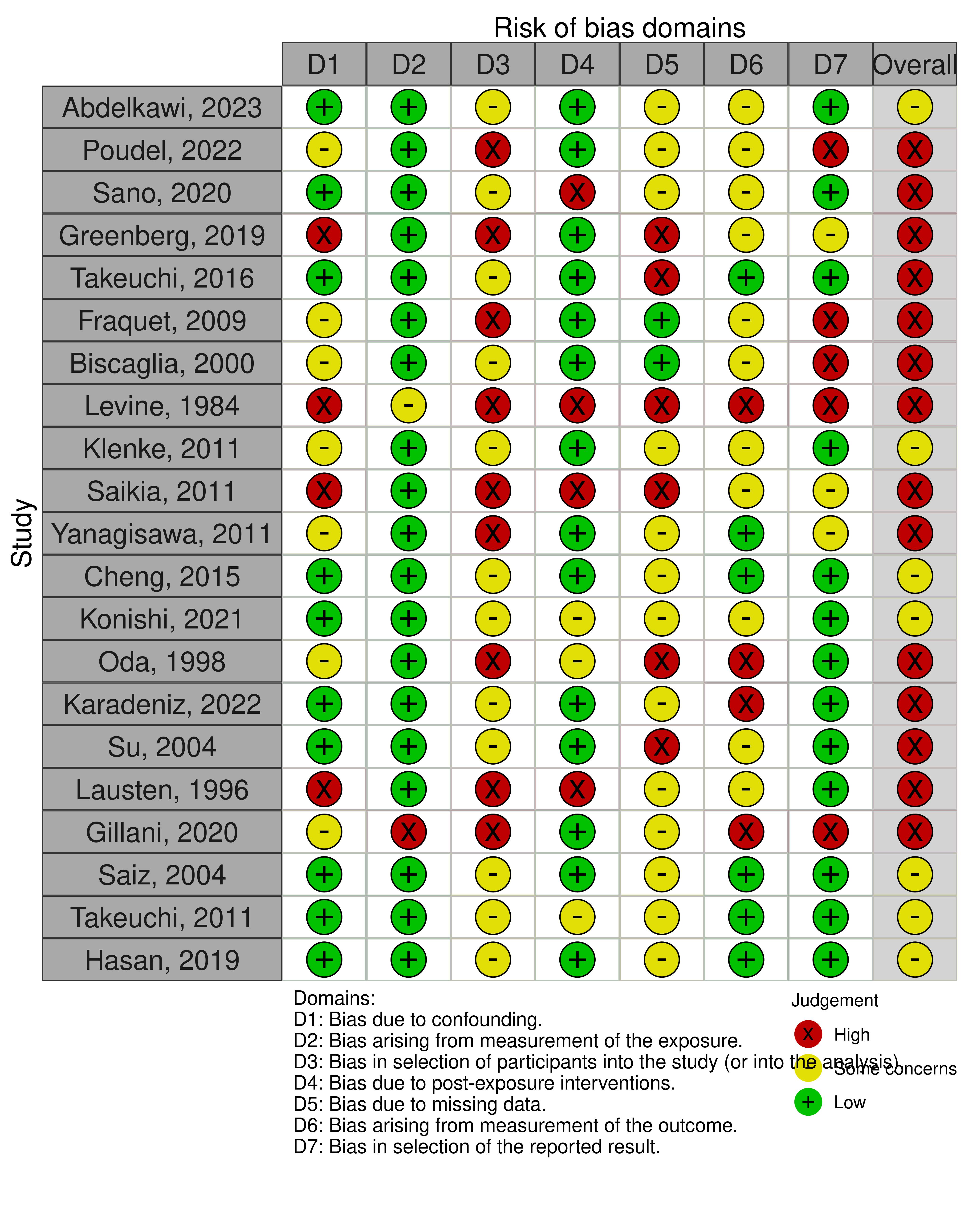
**

1. Risk of bias using ROBINS-I Tool for Interventional studies

**
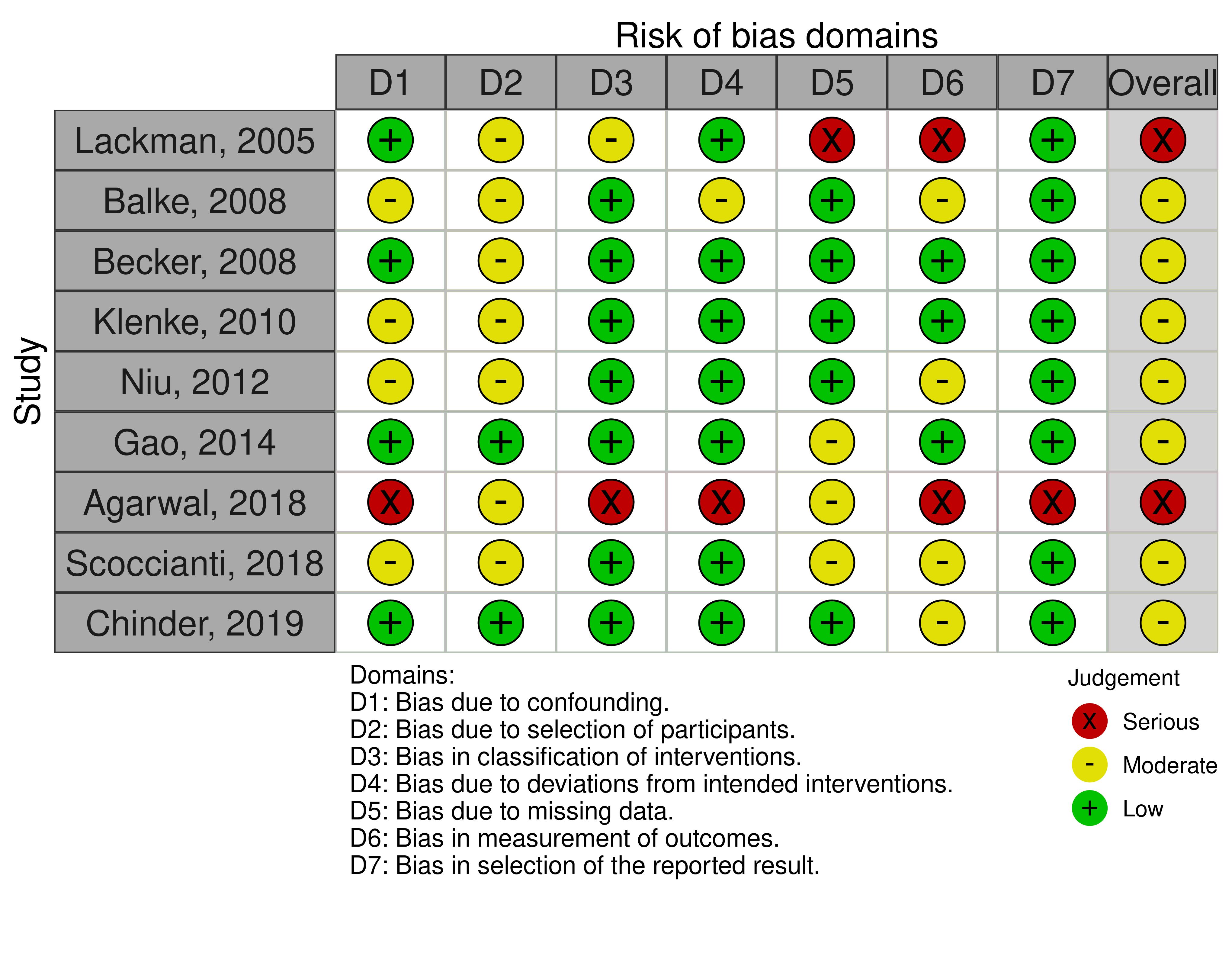
**
